# An assessment of canine ectoparasiticide administration compliance in the USA

**DOI:** 10.1186/s13071-021-05134-1

**Published:** 2022-01-21

**Authors:** Robert Lavan, Dorothy Normile, Imran Husain, Amita Singh, Rob Armstrong, Kathleen Heaney

**Affiliations:** 1 Center for Observational and Real-World Evidence, Merck & Company, Incorporated, Kenilworth, NJ USA; 2 Merck Animal Health, 2 Giralda Farms, Madison, NJ USA; 3 Celeritas Solutions, Limited Liability Company, 157 Columbus Avenue, 4th Floor, New York, NY USA; 4 Zickin School of Business, Baruch College CUNY, 55 Lexington Avenue, New York, NY USA; 5 Heaney Veterinary Consulting, Limited Liability Company, Bradley Beach, NJ USA

**Keywords:** Adherence, Dog, Dosing gap, Ectoparasiticide, Doses plus gap period, Purchase gap

## Abstract

**Background:**

This study evaluated the timing of dog owner ectoparasiticide purchases to estimate administration compliance and assess the consequent impact of dose purchase gaps on the proportion of time that dogs were protected over a 12-month period.

**Methods:**

Ectoparasiticide purchase transactions over a 12-month period were evaluated for 626 US veterinary hospitals to determine dose purchase timing and identify consequent gaps between dose administration in dogs. Orally administered prescription ectoparasitic medications with active ingredients from the isoxazoline family (afoxolaner, fluralaner, lotilaner, or sarolaner) were included in the analysis. A period was calculated for each of the four isoxazoline-containing medications that represented the duration of protection provided by two doses of ectoparasiticide plus the average gap between these two doses. The maximum percentage of time possible for ectoparasiticide protection for this aggregate period was then calculated for each active ingredient.

**Results:**

Ectoparasiticide transaction records of owners were analyzed for 506,637 dogs. These showed that 43% of dog owners purchased just one dose over the 12-month period considered. If a dog owner purchased more than one dose, then the timing of their transactions could create a time gap between the completion of ectoparasite protection from the first dose and onset of protection from the subsequent purchase and administration of the second dose. Such gaps were observed in purchases made by 31–65% of dog owners, depending on the selected active ingredient and number of doses. The average gap duration between dose purchases was calculated for all possible dose combinations over 12 months of ectoparasite protection. Time gaps between the first and second doses are as follows: for sarolaner, 20.3 weeks; for afoxolaner, 12.9 weeks; for fluralaner ,12.8 weeks; and for lotilaner, 8.9 weeks. The proportion of time when protection was provided during the aggregate period between administration of the first and second doses was as follows: for fluralaner, 65%; for lotilaner, 49%; for afoxolaner, 40%; and for sarolaner, 30%.

**Conclusions:**

Dog owner ectoparasiticide purchase transactions showed that there were time gaps between doses leading to reduced ectoparasite protection. The longer re-administration interval for fluralaner, a consequence of its extended duration of activity, resulted in dog owners gaining the greatest proportion of ectoparasite protection time with this medication compared with shorter-acting monthly re-treatment medications.

**Graphical Abstract:**

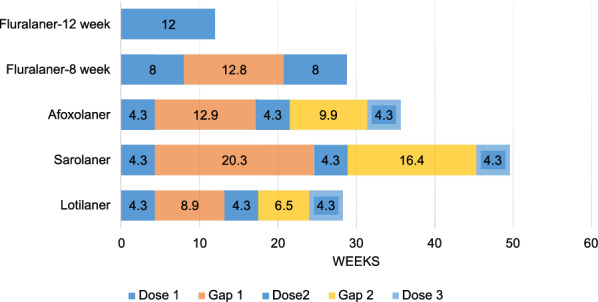

## Background

Ectoparasites, most commonly *Ctenocephalides felis felis* and *Ctenocephalides canis*, and multiple tick species are a common problem for dogs worldwide [1–5]. These ectoparasites cause discomfort, blood loss and can transfer canine vector-borne pathogens, which can also be zoonoses [6–13]. These parasitic vectors are active all year, even in temperate climates, and are increasing their range and frequency of occurrence [14–18]. Many veterinarians and veterinary organizations recommend flea and tick preventive medication administration to dogs and cats year-round [1, 2, 4, 9, 19, 20].

Pet owners are concerned about ectoparasites and vector-borne pathogens, as indicated by ever-increasing online searches for relevant information [6, 21]. Yearly, pet owners worldwide spend a total of 5.2 billion US $ on ectoparasiticidal products to protect their pets from these pests [22]. Many of these products are highly effective against fleas, ticks, and other ectoparasites when administered correctly with re-administration according to the recommended treatment schedule based on their duration of action [23–31]. Unfortunately, pet owners often fail to re-administer ectoparasiticides according to the label recommended re-treatment schedule, as has been evidenced by their failure to purchase sufficient ectoparasiticide doses to adhere to veterinarian recommendations, either continuously throughout the year or seasonally [20, 32–35]. In a survey of 30,020 dogs in Spain, the number of months of flea and tick protection purchased annually by their owners was just 2.9–4.3 months, depending on the product purchased [32]. In another survey of 231,565 dogs in the USA, annual monthly purchases of flea and tick products by their owners were only sufficient for 3.3–5.7 months of protection [33]. These studies clearly show that dog owners fall short in purchasing the quantities of flea and tick medication necessary to meet veterinarian recommendations for effective flea and tick control [32, 33]. However, these studies did not report the timing of dog owner ectoparasiticide purchases or dates of administration. Yet, timely and regular administration of ectoparasiticide medication in a manner consistent with veterinarian recommendations and package insert directions is essential for optimal efficacy and effective ectoparasite control [12, 36–38]. The timely administration of ectoparasiticides doses is key to successful ectoparasite elimination [8, 12, 36–38]. Studies have demonstrated that 2–3 months of continual ectoparasiticides treatment is needed to eliminate a flea infestation [25–27, 31, 38]. Delayed doses or missed doses can interrupt the delivery of continuous ectoparasiticidal therapy, allowing the ectoparasite population to rebound in the home environment. To the pet owner, this rebound may appear as a rapid re-infestation and may cause them to question a product’s efficacy as well as their veterinarian’s recommendation.

Gaps in time between the administration of ectoparasiticide doses can result when either the ectoparasiticidal product is purchased in more than one transaction per year or when the pet owner delays administration of subsequent doses beyond the expiration of the period of efficacy of the previous dose. Unfortunately, there is no easy way to accurately assess home medication administration in a large population of pet owners. Transaction analysis does allow an estimation of the best case administration of medications by assuming that the medication was given on the date of purchase.

The aim of this study was to use a large transaction database to evaluate dog owner ectoparasiticide purchase timing and estimate adherence with veterinary recommendations for flea and tick protection given to dogs. The total number of doses and months of protection purchased in a 12-month period were examined, as well as the timing of those purchases, to allow estimation of the proportion of time that owners provide their dogs with ectoparasiticide protection. Ectoparasiticide purchases and time gaps between these purchases can provide insight into these parameters. A secondary aim was to compare the impact of ectoparasiticide purchase gaps when an ectoparasiticide with a longer, 12-week duration (Bravecto; Merck Animal Health, Madison, NJ) is prescribed compared with monthly duration ectoparasiticides.

## Methods

Dog owner ectoparasiticide transaction records from US veterinary hospitals were analyzed to determine purchase intervals and calculate time gaps between dose purchases. Time gaps between ectoparasiticide dose purchases were used to calculated timely medication administration. A purchase gap was declared when the second or subsequent dose of medication was purchased at a time after completion of the recommended duration of efficacy of the first dose, as described in the package insert. The duration of efficacy was considered to be 4.3 weeks for products intended to be re-applied monthly and 12 weeks for fluralaner. Dog owners were assumed to have administered medication on the date of purchase, therefore, the calculated gap represents the smallest potential time gap between administered doses. A measurable time gap can be determined when pet owners purchase medication in two or more transactions per year. The gap could not be measured between administered doses when the dog owner either purchased multiple doses at the same time or purchased one or more subsequent doses before the conclusion of the period of efficacy of the previous dose. The data were analyzed to calculate the proportion of dog owners who purchased a single dose per year and the proportion who purchased multiple (> 1) doses per year, with a detectable time gap between doses.

Transaction data from 626 veterinary hospitals throughout the USA were examined retrospectively for ectoparasiticides purchased for individual dogs over 6 months of age from 1 January 2017 up to and including 31 December 2019. The lower age limit was selected based on the minimum dosing age in the product labels. Data records were masked to conceal the identity of both the veterinary hospital and the dog owner by using unique numeric identifiers for the dog that allowed each record to be associated with an individual animal. Dog demographic data, including age and body weight, were also collected and summarized. The purchases comprised those of four different prescription isoxazoline ectoparasiticide medications: afoxolaner (NexGard® Chewables; Boehringer Ingelheim Animal Health USA, Duluth, GA); fluralaner (BRAVECTO® Chew; Merck Animal Health, Madison, NJ); lotilaner (Credelio® Chewable Tablets; Elanco, Greenfield, IN) and sarolaner (Simparica® Chewables; Zoetis, Kalamazoo, MI). Because two of these products are indicated for use in dogs 6 months of age and older, data from dogs less than 6 months of age were excluded, and only transactions for dogs that were 6 months of age and older were considered.

To permit comparative analysis, client transaction data were limited to those of owners who stayed with the same brand over the 12-month period. Each transaction record included the date, product—including presentation—and the quantity purchased. Presentations could include single packs, single doses, multi-packs, and/or multiple doses for each product. The transaction records included were for ectoparasite medication sales made by the hospital to the client in the name of a single patient.

The study period for each client was defined as the 12 months following their initial purchase of an ectoparasiticide. Dogs were included if purchase records were available for the 12 months following the initial purchase, regardless of whether more product was purchased or not. Any doses returned to the hospital for credit were excluded. The re-treatment interval for each ectoparasiticide was used to calculate the gaps based on 12-week dosing for fluralaner and monthly (4.3 weeks) dosing for the three other medications. A maximum of 12 purchases was allowed for monthly dosed products and a maximum of five purchases for fluralaner, for administration within a single 12-month period. The label for fluralaner also specifies an 8-week dosing interval for protection against *Amblyomma americanum*, which requires a maximum of seven purchases for 12 months of protection. Each transaction could include the purchase of one or more doses for each product. Because of the purchase gap, each pair of transactions potentially resulted in variable durations of ectoparasiticide protection. Following completion of the protection period of the first dose, there was the possibility of a period of time before the dog owner purchased the next dose. Gaps in protection were calculated based on the purchase dates for each product dose. For a purchase of a single dose, the purchase date was also the “administration” date for that dose, with a protection end date calculated based on the product label recommended re-administration interval. For a single purchase of multiple doses, the administration date of the first dose was the purchase date, and the administration dates for the remaining doses were set based on the recommended re-administration interval, with the assumption that doses were administered consecutively when due. For dog owners with multiple purchase records per year, potentially with a variable number of doses at each purchase, the same assumptions were applied to each purchase record. This analysis assumes that each dose was administered to the dog on the date of purchase and at the correct consecutive interval(s) when multiple doses were purchased. This assumption provides an optimal estimate of timely re-administration of ectoparasiticide doses. If a dog owner deferred giving either the first or subsequent dose(s), then the gap could have been larger than calculated in this analysis.

The total doses purchased were determined for each dog for the entire 12-month period, and the gaps between dose administrations were calculated based on purchase dates. These data were used to prepare a matrix that captured all possible dose-gap combinations. For example, dogs treated with fluralaner could have up to five to seven doses administered within 12 months, and therefore all possible gaps included dose one to two, dose two to three, dose three to four and dose four to five. Dogs treated with monthly dose products could have up to 12 doses administered, and all possible dose gaps could include dose one to two, dose two to three, and so on up to dose 11 to 12. The dose gaps for each possible interval were combined across all of the annual purchased doses for each active ingredient, and were then used to calculate the average gap in weeks for each dose interval.

A Doses plus gap period was created for each two-dose period, and defined as the time duration encompassing the ectoparasite protection interval for the two doses plus the average gap between these doses. The percentage of time when ectoparasite protection was available could then be calculated for each Doses plus gap using the recommended redosing interval for each product (8 or 12 weeks for fluralaner, and 4.3 weeks for the other actives). Dogs were considered unprotected against ectoparasites during the gap portion of time.

Finally, statistical summaries were created for the age and weight of patients within the products being compared. Mean, range, and SEM for age and weight of patient dogs were calculated within the products being compared. Means were compared across groups using a* t*-test with significance set at *P* < 0.05.

## Results

Ectoparasiticide transaction records for four prescription flea and tick products were obtained from veterinary hospitals from across the USA (Table 1). Participating hospitals were located in the southeast (*n* = 345; 55%), mid-west (*n* = 110; 18%), southcentral (*n* = 96; 15%), west (*n* = 39; 6%) and northeast (*n* = 36; 6%) regions of the USA.

**Table 1:** Prescription flea and tick products identified from transaction records obtained in a study of gaps in dose administration

Brand name	Manufacturer	Active ingredients	Indications		Redosing interval
			Fleas	Ticks	
BRAVECTO® Chews	Merck Animal Health	Fluralaner	X	X	8–12 Weeks^a^
Credelio®	Elanco Animal Health	Lotilaner	X	X	Monthly
NexGard® Chewables	Boehringer Ingelheim	Afoxolaner	X	X	Monthly
Simparica® Chewables	Zoetis	Sarolaner	X	X	Monthly

^a^BRAVECTO Chews are labeled for 12-week redosing for most indications, with 8-week redosing indicated when necessary for *Amblyomma americanum*

Dogs ranged from 6 months to 20 years of age, with an average overall age of 6.9 years; their weight ranged from 1.3 to 60.0 kg, with an overall average weight of 18.2 kg (Table 2). Because of the large number of dogs in each group, all means for weight were significantly different from each other [NexGard vs Bravecto, *t*_(240,658)_ = − 33.46, *P* = 3.200E−245; NexGard vs Credelio, *t*_(14,351)_ = 17.57, *P* = 1.06E−68; NexGard vs. Simparica, *t*_(84,486)_ = − 16.79, *P* = 1.88E−63; Bravecto vs. Credelio, *t*_(15,601)_ = 33.16, *P* = 2.11E−233; Bravecto vs Simparica, *t*_(97,805)_ = 8.12, *P* = 2.27E−16; Credelio vs. Simparica, *t*_(20,805)_ = − 25.69, *P* = 1.36E−143]. Similarly, all means for age were significantly different from each other [NexGard vs Bravecto, *t*_(419,183)_ = −74.36, *P* < 0.00001; NexGard vs Credelio, *t*_(19,120)_ = 56.73, *P* < 0.00001; NexGard vs. Simparica, *t*_(119,071)_ = − 27.71, *P* = 5.86E−157; Bravecto vs. Credelio, *t*_(19,841)_ = 85.98, *P* < 0.00001; Bravecto vs Simparica, *t*_(130,112)_ = 27.86, *P* = 1.35E−170; Credelio vs. Simparica, *t*_(25,112)_ = −  66.30, *P* < 0.00001)]. Dogs receiving lotilaner had a mean age approximately 2.4 years younger than the dogs receiving the other isoxazoline products, and a mean weight that was approximately 2.7 pounds (15%) lighter. The age distribution was examined with frequency tables for all flea and tick brands. Each curve was unimodal with a peak at around 2–4 years of age, and a left skew as there were fewer dogs at the higher ages. Given the large sample size, we know from the central limit theorem that, even if the underlying distribution is not normal for sufficiently large sample sizes (*n* usually greater than 30), the distribution of means is normal. The data were continuous, and the sample sizes very large (Bravecto,* n* = 170,792; Credilio, *n* = 16,536; NexGard, *n* = 248,393; Simparica, *n* = 70,916), hence a *t*-test was considered appropriate to determine significant differences between the means.

**Table 2:** Age and weight (*wt*) of dogs identified through owner purchase transaction records

	Afoxolaner (*n* = 248,393)	Fluralaner (*n* = 170,792)	Lotilaner (*n* = 16,536)	Sarolaner (*n* = 70,916)	All dogs^a^ (*n* = 506,637)
Mean age (SEM)	6.6 (0.01)	7.6 (0.01)	4.7 (0.03)	7.0 (0.02)	6.9 (0.01)
Median age (range)	6.4 (0.5–20.0)	6.9 (0.5–20.0)	2.5 (0.5–19.8)	6.3 (0.5–20.0)	6.1 (0.5–20.0)
Mean wt (SEM)	17.6 (0.03)	19.2 (0.04)	15.8 (0.09)	18.7 (0.06)	18.2 (0.02)
Median wt (range)	14.5 (1.8–55.0)	17.6 (2.0–55.9)	13.1 (2.8–45.5)	16.5 (1.3–60.0)	15.7 (1.3–59.9)

^a^Group mean ages and wts of dogs are significantly different for all brands (*t*-test, *P* < 0.0001)

More than half of the dog owners purchased only 1–3 months of flea and tick protection, less than 1/3 purchased 4–6 months of protection, and less than 20% purchased 7–12 months of protection (Tables 3, 4). These ectoparasiticide purchasing records are inconsistent with veterinarian recommendations for nearly year-round flea and tick protection [1, 2, 4, 9, 19, 20].

**Table 3:** Dog ectoparasite protection intervals by product based on doses purchased by the dog owner recorded in veterinary hospital transaction records

Flea and tick protection duration purchased within 12 months	Afoxolaner (*n* = 248,393) (%)	Fluralaner (*n* = 170,792) (%)	Lotilaner (*n* = 16,536 (%)	Sarolaner (*n* = 70,916) (%)
1–6 Months/year	87.0	81.8	88.4	83.7
1–3 Months/year	66.5	54.0	70.0	57.3
4–6 Months/year	20.5	27.8	18.4	26.4
7–12 Months/year	13.0	18.2	11.6	16.3

**Table 4:** Dog owner ectoparasiticide purchases with and without gaps, including average gap duration between doses

Doses purchased	Fluralaner^a^ (*n* = 170,792)	Afoxolaner (*n* = 248,393)	Sarolaner (*n* = 70,916)	Lotilaner (*n* = 16,536)
One dose (Total *n*, % of total)	92,153 (54%)	96,151 (39%)	22,416 (32%)	7401 (45%)
Two to 12 doses (%)	(46%)	(61%)	(68%)	(55%)
Total purchasing > 1 dose	78,639	152,242	48,500	9135
No gap (*n*, %)	27,435 (35%)	104,945 (69%)	28,078 (58%)	5587 (61%)
Purchase gap (*n*, %)	51,204 (65%)	47,297 (31%)	20,422 (42%)	3548 (39%)
Purchased ≥ 2 doses	78,639	152,242	48,500	9135
Total with 1–2 dose gap (%)	42,638 (54%)	28,527 (19%)	11,049 (23%)	2041 (22%)
Average gap (weeks)	12.8	12.9	20.3	8.9
Purchased ≥ 3 doses	31,241	111,420	39,695	6426
Total with 2–3 dose gap (*n*, %)	17,543 (56%)	11,753 (11%)	6040 (15%)	1214 (19%)
Average gap (weeks)	8.5	9.9	16.4	6.5
Purchased ≥ 4 doses	11,742	81,992	29,184	4943
Total with 3–4 dose gap (*n*, %)	4206 (36%)	12,100 (15%)	5641 (19%)	731 (15%)
Average gap (weeks)	4.8	10.1	17.6	6.4
Purchased ≥ 5 doses	1631	69,702	25,583	4271
Total with 4–5 dose gap (*n*, %)	457 (28%)	4389 (6%)	2416 (9%)	436 (10%)
Average gap (weeks)	3.3	7.0	12.9	4.4
Purchased ≥ 6 doses		65,205	23,721	3912
Total with 5–6 dose gap (*n*, %)		2455 (4%)	1344 (6%)	230 (6%)
Average gap (weeks)		5.7	10.7	4.0
Purchased ≥ 7 doses		31,598	10,020	1862
Total with 6–7 dose gap (*n*, %)		5868 (19%)	4187 (42%)	421 (23%)
Average gap (weeks)		9.1	19.4	9.8
Purchased ≥ 8 doses		22,302	7136	1416
Total with 7–8 dose gap (*n*, %)		1246 (6%)	608 (9%)	114 (8%)
Average gap (weeks)		4.3	8.8	3.9
Purchased ≥ 9 doses		18,415	5876	1172
Total with 8–9 dose gap (*n*, %)		703 (4%)	334 (6%)	62 (5%)
Average gap (weeks)		3.4	8.6	3.1
Purchased ≥ 10 doses		14,734	4495	972
Total with 9–10 dose gap (*n*, %)		510 (4%)	332 (7%)	35 (4%)
Average gap (weeks)		3.4	10.3	4.0
Purchased ≥ 11 doses		13,213	3892	865
Total with 10–11 dose gap (*n*, %)		137 (1%)	74 (2%)	14 (2%)
Average gap (weeks)		2.3	6.9	2.6
Purchased 12 doses		12,044	3478	770
Total with 11–12 dose gap (*n*, %)		32 (0.3%)	16 (0.5%)	7 (1%)
Average gap (weeks)		2.1	3.6	1.6

^a^The dosing interval for fluralaner is 12 weeks for most parasites, according to the product label; a maximum of five doses of purchased topical fluralaner were considered for the 12-month period studied. (Note: up to seven doses of fluralaner may be required for full-year protection in areas where *Amblyomma americanum* is of concern)

Nearly half (43%) of all the dog owners purchased just one dose of ectoparasiticide in the 12-month period regardless of the flea/tick medication concerned. Similarly, close to half (42%) of the dog owners who bought more than one dose allowed time gaps between their purchases of flea and tick medication (Table 4). The proportion of transactions that created a protection gap tended to decrease with increasing numbers of doses purchased because the average length of these gaps tended to decrease. However, for dog owners who purchased more than six doses of the monthly administered products, the proportion of transactions with gaps increased between doses six and seven (Table 4). The longest average purchase gap duration was calculated for dog owners who bought sarolaner (3.6–20.3 weeks), with shorter gaps for fluralaner (3.3–12.8 weeks), afoxolaner (2.1–12.9 weeks) and lotilaner (1.6–8.9 weeks) (Table 5).

**Table 5:** Proportion of each two-dose plus purchase gap interval with and without ectoparasite protection

Protection duration (nominal months)	Product	Fluralaner^a^ (12-week dosing)		Product	Afoxolaner^b^		Lotilaner^b^		Sarolaner^b^	
	Fluralaner gaps	Doses plus gap period^c^ (weeks)	Percentage of time protected	Monthly product gaps	Doses plus gap period^c^ (weeks)	Percentage of time protected	Doses plus gap period^c^ (weeks)	Percentage of time protected	Doses plus gap period^c^ (weeks)	Percentage of time protected
1	No gap^b^			No Gap						
2				Dose 1–2	21.5	40	17.5	49	28.9	30
3				Dose 2–3	18.5	46	15.1	57	25.0	34
4	1–2 Doses	36.8	65	Dose 3–4	18.7	46	15.0	57	26.2	33
5				Dose 4–5	15.6	55	13.0	66	21.5	40
6				Dose 5–6	14.3	60	12.6	68	19.3	45
7	2–3 Doses	32.5	74	Dose 6–7	17.7	49	18.4	47	28.0	31
8				Dose 7–8	12.9	67	12.5	69	17.4	49
9				Dose 8–9	12.0	72	11.7	74	17.2	50
10	3–4 Doses	28.8	83	Dose 9–10	12.0	72	12.6	68	18.9	46
11				Dose 10–11	10.9	79	11.2	77	15.5	55
12	4–5 Doses^d^	27.3	88	Dose 11–12	10.7	80	10.2	84	12.2	70

^a^For fluralaner, the single dose duration of efficacy was 12 weeks per label indication for most parasites, with no gap

^b^For afoxolaner, lotilaner and sarolaner, the single dose duration of efficacy was 4.3 weeks, with no gap

^c^Doses plus gap period is the duration encompassing the ectoparasite protection interval for the two doses, as indicated by the product label, plus the average gap between the purchase of these two doses

^d^Four to five doses of fluralaner provided ectoparasiticide protection beyond the 12-month study duration

For fluralaner purchased by dog owners, the protection proportion calculated for the Doses plus gap period gradually increased with subsequent doses, which indicated a greater duration of administered protection, as follows: 65% for the period between doses one and two; 74% for the period between doses two and three; 83% for the period between doses three and four; and 88% for the period between doses four and five (Table 5). The protection proportion of each two-dose plus gap period for the monthly administered products was smaller than for fluralaner at the dose intervals for the first six doses (Table 5). The protection proportion for all the monthly medications generally increased, and the gap size decreased from dose periods one to 12, with the exception of dose period six to seven, which was one of the longest gaps between purchases (Table 5).

**Fig. 1:**
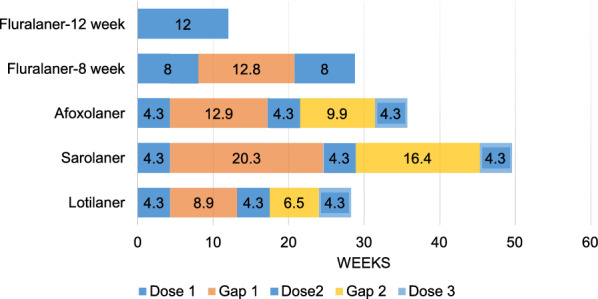
Impact of ectoparasiticide purchase gaps when up to 3 months of ectoparasite protection is purchased with gaps, as determined from veterinary hospital transaction records of dog owners.* Fluralaner-12 week* Fluralaner with a 12-week dosing interval using one dose,* Fluralaner-8 week* fluralaner with an 8-week dosing interval using two doses

**Fig. 2:**
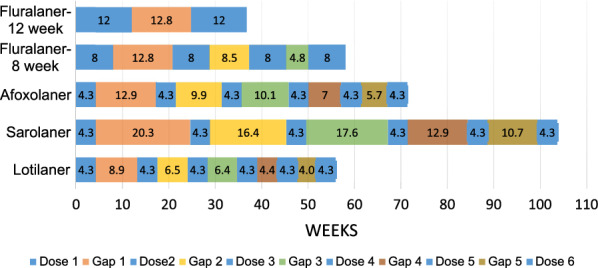
Impact of ectoparasiticide purchase gaps when up to 6 months of ectoparasite protection is purchased with gaps, as determined from dog owner veterinary hospital transaction records. For dosing intervals of fluralaner, see Fig. 1

The impact of purchase gaps on the percentage of time that ectoparasite protection was available is shown for owners who purchased 1–3 months and 1–6 months of flea and tick medication in a year, the most common amounts of protection purchased (Fig. 1 cf Table 6; Fig. 2 cf Table 7). The fluralaner dosing interval is 12 weeks, therefore one and two doses (2.8 months and 5.6 months) of fluralaner were compared to three and six doses of the monthly duration products afoxolaner, sarolaner, and lotilaner. Because fluralaner is approved in the USA for use at an 8-week dosing interval when necessary for the control of *A. americanum*, two and four doses of fluralaner were also compared to three and six doses of the monthly duration products. The total duration of the 3-month and 6-month Doses plus gap period for each product are shown in Figs. 1 and 2, respectively, and for each of these periods, the percentage of time when ectoparasiticide protection could have been available was determined and compared.

**Table 6:** Impact of purchase gaps when up to 3 months of ectoparasite protection is purchased with gaps

Product	Three-month dosing period including average gap duration (weeks)	Percentage of period protected
Fluralaner 12-week dosing^a^	12	100
Fluralaner 8-week dosing^b^	28.8	56
Afoxolaner	35.7	36
Sarolaner	49.6	26
Lotilaner	28.3	46

^a^Fluralaner with a 12-week dosing interval using one dose

^b^Fluralaner with an 8-week dosing interval using two doses

**Table 7:** Impact of purchase gaps when up to 6 months of ectoparasite protection is purchased with gaps

Product	Six-month dosing period including average gap duration (weeks)	Percentage of period protected
Fluralaner 12-week dosing	36.8	65
Fluralaner 8-week dosing	58.1	55
Afoxolaner	71.4	36
Sarolaner	103.6	25
Lotilaner	56.0	46

For dosing intervals of fluralaner, see Table 6

The percentage of ectoparasite protection available during the first 12 weeks or 3 months of the purchased product was 100% for fluralaner with the 12-week dosing interval (12 weeks or 2.8 months) and 56% with the fluralaner 8-week dosing interval, 36% for afoxolaner, 26% for sarolaner, and 46% for lotilaner (Fig. 1). The percentage of ectoparasite protection available when dog owners purchased up to 6 months of medication was 65% for fluralaner with the 12-week dosing interval and 55% with the fluralaner 8-week dosing interval, 36% for afoxolaner, 25% for sarolaner, and 46% for lotilaner (Fig. 2).

## Discussion

Timely and regular administration of ectoparasiticide medication in a manner consistent with veterinarian recommendations and package insert directions is essential for optimal effectiveness and effective ectoparasite control [12, 36–38]. Prior studies have shown that dog owners fall short in purchasing the quantities of flea and tick medication necessary to meet veterinarian recommendations for effective flea and tick control [32, 33]. The present study of ectoparasiticide purchase records for the owners of 506,637 dogs confirms these previous findings, with 43% of dog owners purchasing just one single dose of ectoparasiticide medication in a 12-month period, and 54–70% of dog owners purchasing just 1–3 months of ectoparasiticide protection. The present study also shows that, of dog owners purchasing more than one dose of ectoparasiticide, 42.4% delayed purchasing subsequent doses beyond the efficacy duration of the prior dose noted in the manufacturer’s product insert, making timely redosing impossible.

Delays in timely ectoparasiticide purchases represent periods when dogs may not be protected against fleas and ticks. In addition, if an established ectoparasite population was not eliminated by prior treatment, then a purchase gap interrupts treatment and may allow that parasite population to recover. Also, treatment gaps lead to an increased risk of prolonged ectoparasite infestation and potential exposure to vector-borne pathogens [36, 38].

For this study, medication administration was assumed to have occurred on the day of purchase. The owners may have delayed dose administration for days, weeks, or longer periods following purchase, but the actual day of administration was not verified in this study, which involved a large dog owner population. Therefore, the timing of medication administration presented here represents the best possible (shortest) amount of time between the delivery of consecutive doses. However, studies in both human and veterinary medicine have shown that timely administration frequently does not occur, even when all necessary doses are dispensed at once [35, 39–44]. Therefore, it is possible that the actual time until administration was longer than reported here, and delays in administration may have occurred even when multiple doses of medication were purchased at one time.

To take into account the realities of dog owners purchasing and administrating ectoparasiticides to their dogs, the present study examined the value of the longer duration fluralaner medication compared to monthly administered flea and tick medications. The benefits come from the length of time that a single fluralaner dose protects against ectoparasites as well as the reduced number of doses that need to be given to provide protection for a given period of time. The facts that owners allow gaps to occur in their flea and tick medication purchases and that there is a need for more frequent redosing intervals for monthly administered products result in smaller proportions of time when dogs receive ectoparasite protection than is seen with longer duration medication. More frequent gaps in dosing for monthly products can reduce their effectiveness in accomplishing the goal of ectoparasite elimination and consistent ectoparasite control.

When dog owners purchase more than 1 month of flea and tick medication, they often purchase either 3 months or 6 months of protection. If a dog owner’s goal is to provide continuous protection for these 3 or 6 months, then 12 weeks of continuous protection can be provided for their dog with one dose of fluralaner or multiple 1-month periods of protection using shorter duration ectoparasiticides. The shorter duration products require repeated on-time dosing to achieve the same continuous protection as one dose of fluralaner. The gap analysis demonstrates that dog owners buying monthly products usually do not achieve the goal of on-time dosing, with inter-dose intervals that range from 9.9 to 12.9 weeks for afoxolaner, 16.4–20.3 weeks for sarolaner, and 6.5–8.9 weeks for lotilaner. The proportion of time when ectoparasite protection is provided with the purchase of up to 3 months of product are: 100% for fluralaner (2.8 months or 12 weeks), 36% (3 months) for afoxolaner, 26% (3 months) for sarolaner and 46% (3 months) for lotilaner (Fig. 1a). If a shorter 8-week dosing interval is used for the calculation for fluralaner, then the percentage of time when ectoparasite protection is available is 56%.

Gaps in flea and tick protection when dog owners purchase 6 months of annual treatment are shown in Fig. 2. The size of the gap usually shrinks when a dog owner buys more months of protection. This decrease in the gap may simply be secondary to the effect of purchasing more doses within a dwindling period of possible unprotected time. Owners who purchased two doses of fluralaner obtained 24 weeks of protection for their dogs with a 12.8-week gap between doses, resulting in the dogs being protected from fleas and ticks for 65% of the Doses plus gaps interval. The monthly product purchases were spread out by gaps of various sizes that ranged from 4 to 20 weeks between doses, which provided protection from fleas and ticks for 36% of the Doses plus gaps interval for afoxolaner, 25% for sarolaner and 46% for lotilaner. Even if a shorter 8-week dosing interval is used for the calculation for fluralaner, the percentage of the Doses plus gaps time when ectoparasite protection is available is 55%.

For each of the flea and tick medications dosed monthly, the longest Doses plus gap interval was between the sixth and seventh dose (Table 5). Manufacturers often package monthly flea and tick medications into packs of three or six doses. The size of the gap following the sixth dose may be secondary to a delayed repeat purchase following an initial purchase of a single card with six doses.

These differences in number and duration of purchase gaps and the resultant percent of time when dogs are protected against ectoparasites have practical implications. Interruptions in ectoparasiticide protection that arise from either gaps in dose purchase timing or other delays in their administration can result in a perceived lack of effectiveness if the duration of continuous use is insufficient to eliminate established infestations on the animal, in the home, or in the face of continued parasite exposure. Previous studies have shown that multiple, consecutive doses of afoxolaner, sarolaner, or lotilaner were required to eliminate an established flea infestation, with evidence of the infestation continuing following a single dose [23, 26–28]. A treatment gap between doses of a monthly ectoparasiticide may allow the infesting ectoparasite population to rebound if the population is not eliminated. The longer duration medication, fluralaner, has been demonstrated to provide ectoparasiticide protection for a duration that provides complete resolution of flea infestation without redosing [26]. Longer treatment periods may be required to eliminate a flea infestation, particularly where other animals or sources of re-infestation are present.

Studies on patient and pet owner adherence to prescribed treatment regimens in human and veterinary medicine have shown that simpler, less frequent dosing regimens improve compliance across a variety of therapeutic classes [39–49]. The present study similarly demonstrates the benefit of less frequent dosing with a longer duration ectoparasiticide. Dog owners who purchased ectoparasiticides with time gaps between doses, but chose the longer duration product, provided more consecutive weeks of medication, reduced treatment interruptions, and increased the overall percentage of time when ectoparasite protection was available during each Doses plus gap period than dog owners who purchased a monthly treatment. Such increases in the overall duration of ectoparasite protection with fewer interruptions provided by the longer duration fluralaner medication should improve ectoparasite control, decrease exposure to vector-borne pathogens and provide greater pet owner satisfaction in their efforts to remove ectoparasites from their dogs.

In this study, dogs receiving lotilaner were, surprisingly, younger and lighter in body weight than dogs receiving the other three isoxazoline flea and tick products. We believe these differences are related to the 3-year time period (2017–2019) during which the data were collected. Bravecto, Simparica and NexGard were introduced into the US market before 2017. Lotilaner was introduced in 2018, thus its market was just building during the time that the present study was carried out. We believe that the new prescriptions for lotilaner were for younger dogs and puppies that were starting to receive flea and tick medication, rather than for older dogs for whom owners were switching medication from one isoxazoline product to another. In time, we would expect dogs receiving lotilaner to approximate the body weight and age of the dogs receiving the other isoxazoline products.

In the USA, dog owners purchase isoxazoline ectoparasiticides with a prescription from a veterinarian. We have seen that veterinarians may recommend up to 12 consecutive months of protection for fleas and ticks. A dog owner has the option to purchase one or multiple doses through one or more transactions. Veterinarians expect dog owners to use the doses that they purchase, and that they will give the next dose to their pet when the label-recommended protection interval of the previous dose has been completed. More information is still needed to establish actual owner behavior in the administering of flea and tick medications at home; however, based on veterinary hospital transaction records, it is clear that there are delays in ectoparasiticide administration. Because of the relative regionality of the data (i.e. 55% from the southeast), it is not known if these purchase patterns and dosing gaps are representative for the entire USA or for other countries.

The veterinarian has several options to increase pet owner compliance with flea and tick prevention recommendations, including financial incentives (coupons, discounts), reminder systems that message the owners on their smartphones, increasing the amount of parasite education, and the use of extended-duration parasite control products. There is a real benefit to a pet owner and their dog when a longer-acting ectoparasiticide is prescribed, which is directly related to the additional weeks/months of coverage per administered dose and the need for fewer dose administrations throughout the year.

## Conclusions

This study demonstrated that dog owners fail to adhere to veterinarian recommendations for ectoparasiticide protection by (i) purchasing fewer doses of ectoparasiticide than recommended, and (ii) allowing gaps between dose purchases that indicate a lack of adherence to recommended re-administration intervals. The dog owners obtained more consecutive weeks of ectoparasite protection and a larger overall percentage of time of protection for their pets when a longer duration medication, fluralaner, was prescribed compared with medications that are re-administered monthly.

## Data Availability

The datasets were obtained from a public source (VetInformatics, Rolling Meadows, IL). The data analysis was generated during the current study and is not publicly available because it is the proprietary property of Merck & Company, Incorporated, Kenilworth, NJ.
